# The Insurance Bill and the Hospitals

**Published:** 1911-12-16

**Authors:** Howard J. Collins

**Affiliations:** (House Governor). General Hospital, Birmingham.


### THE INSURANCE BILL AND THE HOSPITALS.

*To the Editor of* The Hospital.

Dear Sir,—It seems almost useless to repeat the apprehension of all hospital authorities of the disastrous risk of the National Insurance Bill as at present drawn, unless some of the amendments which have been passed *en bloc* on the report stage, have entirely altered this aspect of the Bill. That the very existence of voluntary hospitals is jeopardised seems to be clear, unless the very sweeping suggestion of the Chancellor of the Exchequer himself is to be adopted—that in future no insured person can be received at a voluntary hospital—and this seems to be extremely hard on the working classes, who will have compulsorily to be insured and will then not be provided for in any really serious condition. That the Bill may have some effect in enabling hospitals to decline a number of applicants in the out-patient department seems probable, but on the other hand the pressure on the beds will increase rather than diminish, and for provincial hospitals at any rate the freedom with which serious cases have been admitted must be restricted.

It will clearly be difficult for hospital committees to reconcile the treatment of insured persons under a State scheme, with the present fundamental principle of treating in voluntary hospitals those who are unable to provide the necessary treatment, and if the Chancellor of the Exchequer's advice is generally accepted in this matter, the effect on the contributions of the well-to-do and philanthropic public must be great, while the large mass of contributors of one and two guineas a year will naturally hesitate in view of the increased amount they would have to pay for their servants or other employés.

If some 30 per cent. of the present income (as is estimated) may be affected, it must be a very serious question as to the maintenance of the full number of beds, for which at present the income is not sufficient.—Yours truly,

Howard J. Collins

General Hospital, Birmingham. (House Governor).

December 5.

